# Monthly Summary

**Published:** 1868-04

**Authors:** 


### MONTHLY SUMMARY.

*Surgical Notes.*—By Theo. A. M'Graw, M.D., Attending Surgeon to Harper Hospital.—J. W., a Scotchman, of 35 years of age, consulted me on November 19th, about a disease of the bones of the face, from which he had suffered for about two years. He had received a blow across his nose from a falling bough, in the winter of 1865, and shortly after, in his business as a lumberman, was exposed to such a degree of cold as to make him think that he had actually frozen his head. From that time forward, he had been suffering more or less from a foul discharge from the nose, had lost a part of the alveolar process of his upper jaw, and had experienced difficulty in swallowing. Under the treatment of various physicians, he had at times been relieved of some of his most troublesome symptoms, but never cured of the disease. A month previous to his visit to Detroit, he had noticed a gradually increasing redness of the skin of his nose, and finally had become much alarmed at the appearance of an ulcer, through which pus discharged from the nasal cavities. He denied very positively having ever had any venereal disease whatever, but said he had at one time suffered for years from a chronic ulcer of the leg. On examining him, I found a defect in the alveolar process of the upper jaw, corresponding to the eye-tooth and first molar of the right side. There was a hole about as large as a quill in the soft palate. He said that it had been much larger, but had gradually contracted to its present dimensions. The skin on the bridge of the nose was ulcerated through, exposing the nasal bones, the opening being about two lines in diameter. His appetite was good and his bowels regular, though the discharge from his nose was of such an offensive, fetid nature, as to sicken everybody about him. As the ulcer was rapidly extending, and threatened to destroy the nose, I did not think it wise to delay the operation, though doubtful whether I should be able to remove the whole of the diseased tissues. Accordingly, on November 20th, assisted by Dr. Walker and Mr. McGillycuddy, a student in my office, I made a transverse incision through the integument, the cut passing directly across the ulcerous opening. Having removed the necrosed nasal bones, I inserted my forefinger through the aperture, in order to learn the extent of the disease. I found that the nasal processes of both superior maxillary bones, the ethmoid, the nasal spine of the frontal bone, all the turbinated bones, and the rostrum of the sphenoid bone, were all involved in the morbid process. The vomer had completely disappeared. With a pair of bone forceps and a gouge, I was enabled to remove the greater part of the diseased tissue, but was obliged to leave the cribriform plate of the ethmoid, though probably involved in the disorder, and part of the rostrum, the latter being yet firmly attached to the sound bone, and being difficult of access on account of its position. The soft parts were then drawn together, and fastened with sutures, though from their inflamed condition and the bruising which could not be avoided in the operation, I did not entertain much hope of their healing by first intention. The nostrils were syringed with a weak solution of liq. sodæ chlorinatæ several times a day. On the third day after the operation, the stitches were removed, and the parts held in apposition by adhesive plaster. Union took place, however, very slowly, and at one time the surrounding tissues became so much inflamed that I was fearful lest they should slough. The application of sedative solutions of acetate of lead and sulphate of zinc seemed to have little effect. The wound improved somewhat after being touched with a stick of nitrate of silver, but was most favorably affected by the unguentum plumbi iodidi, which I was finally induced to apply on the recommendation of Dr. Brodie. It was not, however, until the middle of December that he had sufficiently recovered to resume his usual avocation. His nose, from a rather large Roman, had, by the removal of its bony support, been changed into a not very handsome pug; but is capable of improvement by a plastic operation at some future time, when the disease shall have entirely vanished. He has still a slight discharge, for the correction of which he is to inject a weak solution of liq. sodæ chlorinatæ (ʒ i to aqua ℥ i), but is otherwise in good health.

In this case, the affection of the bones was undoubtedly of constitutional origin, though the blow on his nose and exposure to cold may have been the exciting causes. The chronic ulcer of the leg, from which he had previously suffered, was doubtless an indication of some constitutional malady, whether scrofulous or syphilitic I could not determine. I put him on a course of iodide of potassium, ten grains three times a day, in order to meet either disease.

It is to be remarked, that besides the necrosis which existed in continuity, the patient had lost a part of the alveolar process of the upper jaw, though the bone intervening between it and the disease in the nasal cavities was sound.—*Detroit Review of Medicine and Pharmacy.*

---

*Poisonous Hair Washes.*—The *Journal of Applied Chemistry*, has the following pertinent remarks on this topic:

"That there is a great need of better information among the common classes regarding the poisonous compounds sold under thousands of different names as hair washes, hair invigorators, and hair dyes, is constantly pressed upon our attention from many sources. Even those who should be better informed seem to be so generally thoughtless or criminally neglectful that the importance of the subject is almost totally lost sight of, unless public attention happens to be called to it by a striking example of the evil effects of those poisons. The following from the *Utica Observer*, is worth the attention of all who use any sort of dyes or artificial "restoratives" for the hair:

"The *Journal of Applied Chemistry*, for this month, asserts that paralysis has been produced by the use of lead, employed to give a dark color to hair, and says the various 'hair restoratives' so much in vogue, which contain sugar of lead, should be forever abandoned. I am induced to send to you this note from the fact that a lady recently called on me for advice in a case of paralysis of the left eyelid and tongue, caused entirely by the use of a popular hair nostrum.

She was recommended to a skillful practitioner, who will probably find great difficulty in curing the malady for it is notorious that lead is one of the most difficult poisons to expel from the human system.

But, sir, although not competent to prescribe for this rapidly increasing malady, it is easy enough to give the method for detecting the lurking fiend, which is to take one grain of iodide of potassium and drop it in a three ounce bottle of the suspected stuff; shake it slowly for a minute, and if the compound turns yellow and ropy, empty the contents of the bottle into the cesspool, no matter what it cost or by what balsamic name it is known in the advertising column."

We thank our correspondent for his communication. It touches upon a subject of more importance than the public are generally aware of. The hair restoratives containing sugar of lead are doing the world harm, and the law should stop their manufacture and sale. One who is very dear to the writer of this paragraph has been for years an invalid, from the use, as physicians and herself are fully convinced, of a hair dye which was formerly very popular and much used. The encroachments of disease caused by the preparation referred to began with a gradual loss of power in the left arm, and thence the weakness has extended until nearly the whole system has become paralyzed. For two years the patient has not been able to leave her bed, and for nearly half that period she has been unable to speak! To nothing else than the use of hair restoratives containing sugar of lead is her present condition to be attributed. But for that she would probably be well, strong, and useful today.

We take blame to ourselves for having failed to speak strongly and often on this subject before now, and say to every one who reads this: use no hair dye which has not been carefully analyzed, and which your physician can not certify to you to be harmless. The cases are numerous wherein partial or total paralysis has resulted from the application of the villainous compounds of which we are speaking.

---

*Carbolated Glycerine.*—By George W. Lawrence, M.D., of Hot Springs, Arkansas.—Carbolic acid is an agent that justly merits universal medical attention. It is not my purpose to enter into its interesting progressive history, or array the names of those distinguished characters associated with Runge, (the discover,) in its chemical literature. Suffice it to state, that I am too thankful to have carbolic acid in its detached crystals, so separated for me from its prodigious and important relatives, from a family so complex and varied in nature, so mysteriously intimate in congeners, that a complete knowledge of petroleum, its bountiful and wonderful chemical productions, affords alone a theme, as a *speciality* for consideration. My object is simply to bring into general notice and use a preparation, which I have styled, (for the want of a better name,) *Carbolated Glycerin.* It is prepared from Calvert's beautiful crystalline, chemically pure pyrogenous acid—not from the *crude empyreumatic fluid of commerce,* called phenol, phenic acid, carbolic acid, etc. It is the *camphoroid* solid acid that I use in forming the *carbolate*, with PRICE'S or BOWES' inodorous glycerin. In a water bath, ranging from 100° to 130° Fahr't, I mix *one* ounce of carbolic acid (when fluid) with *nine* times (in bulk,) of pure glycerine, and agitate while hot until it is thoroughly incorporated. I find this a convenient strength for dilution when required. With this preparation and its dilutions with glycerine or water, I claim an agent that will relieve and control with more certainty and celerity, phagadena, sloughing ulcers, bed sores, chronic syphilitic, mercurio, syphilitic, and strumous ulcerations, sloughing gummates, phagadenic chancres, and all of that class of obdurate ills, more satisfactorily than any application that has come within the range of my experience. It is beneficial in cutaneous diseases of a *parasatic* origin. Diluted ten to twenty times its bulk *with pure water*, I use it with RICHARDSON'S "*Atomizer*," for all forms of aggravated ulcerated surfaces. With the "*Nephogene*," it is invaluable for nasal, faucial, tonsillar, pharyngeal, laryngeal, tracheal and bronchial ulcerations. With an adaptation I recently had made to the French instrument called the "*Irrigateur*,"—I convey at will the solution of carbolated glycerine to any part of the person. In ulcerations of the uterus and vagina, and in the treatment of follicular discases of the genitals, it is an important agent. For sinuses, ulcerations and fistulous opening in the rectum it is advantageous in its effects. I also use it for ulcerations of the external auditory channel. In caries and necrosis of the bones, wherever it can be applied, I employ with a solution of *chlorate of soda*. As an antiseptic, disinfectant, anti-parisatic, detergent, corrective and healthy stimulant, it is assuredly one of the most powerful and valuable adjuncts to our list of Remedies.—*Med. and. Surg. Reporter.*

---

*Mental and Manual Labor.*—Professor Houghton of Trinity College, Dublin, has published some curious chemical computations respecting the relative amounts of physical exhaustion propuced by mental and manual labor. According to these chemical estimates, two hours of severe mental study abstract from the human system as much vital strength as is taken from it by an entire day of mere hand work. This fact, which seems to rest upon strictly scientific laws, shows that the men who do brain-work should be careful, first, not to overtask themselves by continuous exertion; and, secondly, that they should not omit to take physical exertion, on a portion of each day, sufficient to restore the equilibrium between the nervous and the muscular system.—*Med. and Surg. Reporter.*

---

*Muscle Sugar.*—In Aug. 1861, G. Meissier announced his discovery of a true sugar in muscle. Dr. J. Rauke has reinvestigated the subject and fully confirms Meissier's supposition. The following propositions are considered as established: First. That there exists a true fermentable sugar in muscle. Second. That the amount of this sugar is increased by muscular action, including tetanization caused by strychnia or electricity. Third. That the liver has no effect in causing this increase; for the sugar is proved to arise in the muscle itself, and from the muscular substance.—*Med. and Surg. Reporter.*

---

*A Simple Method of Protecting Water from the Action of Lead Pipe.*—*Dingler's Polytechnisches Journal* publishes a simple method, brought forward by Dr. Schwarz, of Breslau, for preventing the poisonous influence of lead pipes on water, by forming on the inside surface of the pipes an insoluble sulphuret of lead, which has proved so effective that, after simple distillation, no trace of lead can be detected in water which has remained in the pipes for a long time. The operation, which is a very simple one, consists in filling the pipes with a warm and concentrated solution of sulphuret of potassium or sodium; the solution is left in contact with the lead for about fifteen minutes. Commonly, a solution of sulphur in caustic soda will answer the purpose, and produce practically the same results. It is known that sulphuret of lead is the most insoluable of all compounds of lead, and nature itself presents an example which justifies the theory of Dr. Schwarz, since water extracted from the mine of Galena does not contain lead, a fact which has often occasioned surprise.

---

*Nervi Nervorum.*—M. Charles Robin has lately announced to the Academy of Sciences that M. Sappey has discovered the *nervi nervorum*, the existence of which had been previously suspected, although they had never been seen. By the aid of the microscope he has seen them in the neurilemma, the cellular and resisting membrane that forms, about each nerve, as well as about its component nerve fibres, a sort of canal in which is ledged the nervous pulp. They are very small fibres, anostomosing in every direction, and in diameter do not exceed five hundredths of a millimetre.—*Medical Gazette.*

---

*Effects of Alcohol on Men and Animals.*—Experiments made by Drs. Ringer and Rickards on the effects of alcohol on men and animals, go to show that the temperature of the body falls nearly as fast after the use of alcohol in doses sufficient to produce intoxication, as after death itself. The facility with which drunkards freeze to death, is explained by this fact. Dr. Jolly declares, that an increasing tendency towards mental disease has been generated by the increasing consumption of spirits. Official reports show, that the abuse of alcohol accounts for one-fifth of the insanity in France.—*Medical Gazette.*

---

*Nitrous Oxide as an Anaesthetic in Surgery.*—In the first number of the *Continental Gazette*, the new American newspaper just started in Paris, we find an account of the removal of a cancerous breast, by Dr. Marion Sims, in which nitrous oxide gas was used as the anæsthetic, with entire success. The patient was sixty years of age, rather stout, and of lymphatic temperament. Anæsthesia was produced in two minutes, and was kept up for sixteen minutes longer. In less than one minute after inhalation ceased consciousness was complete. There was no nausea or vomiting. The account states that after the patient was first made insensible, she was allowed to breathe some air with the gas, and was thus *returned to semi-consciousness*, and continued in this condition during the entire operation. She declared, after the operation, that while inhaling the gas, she could see Dr. Colton and Dr. Evans, (who administered it,) but felt no pain, though she experienced a kind of pushing sensation.

"There were present to witness the operation Baron Larry, Surgeon-in-chief to the Army; Sir Joseph Oliffe, physician to the British Embassy; Dr. Pratt; Dr. Vanzant; Dr. Pope, of St. Louis; Dr. Stearns, of Boston, U. S.; and some others of note, all of whom united with Dr. Sims in expression of surprise and delight at the operations of this new anæsthetic agent.

This was, perhaps, one of the operations where the patient was kept insensible for the greatest length of time with the gas, and it certainly proved eminently successful."—*Medical Gazette.*

---

*Remarkable.*—At the battle of Williamsburg, in May, 1862, Gen.——, of this place, was dangerously wounded by a minnie ball which went entirely through his face, crushing in its passage his jaw-bone and knocking out several of his jaw-teeth. From this wound he has been a constant sufferer until a few days ago, when he had extracted, from near the root of his tongue, a large jaw-tooth, where it was driven by the ball which wounded him near six years ago.—*Warrenton Sentinal.*

---

*Sloughing Produced by Local Anaesthesia.*—The *Lancet* notices a case of this kind occurring in the Middlesex Hospital, the patient being a young woman. "Mr. Lawson had diagnosticated the existence of an abscess behind the patint's breast, and as the pus was very deep (under the pectoral muscle indeed) the refrigerator was used, paraffine ether being employed. Congelation was rapidly produced, and kept up for a few minutes. The result has been, that a portion of skin, about an inch by three-quarters of an inch, over the upper part of the breast, has sloughed, and its healing will necessarily be attended by an unseemly scar. The case is certainly exceptional; but the circumstance is worth remembering when exposed parts of the body are to be operated upon."

---

*Assimilation of Gelatin.*—Most books on physiology claim that gelatin has no claim to be considered a tissue form. Dr. Charles A. Cameron, in a paper read at the recent meeting of the British Association for the advancement of science, objects to this as being too broad a statement. He points out the fact that this substance does not appear in the fæces, as it should, if it did not enter into the system, but that part of it, at least, passes off through the kidneys as urea. Animals cannot live exclusively upon it. It contains neither sulphur nor phosphorus, elements essential to the nutrition of muscles and nerves. By mixing, however, with gelatin the phosphorized and sulphuretted fats found in the brain and nervous tissue, a food is obtained capable of supporting animal life. Dr. Cameron kept a white mouse in a very healthy state for forty-two days, on a diet of this character.

*Death from Chlorodyne.*—On Saturday last considerable excitement was created in the town of Harleston by the report that a woman named Elizabeth Saunders had been poisoned by chlorodyne. The decease has tor several years been in the employ of Mr. Thomas S. Stanton, of Mendham, who supplies this town with milk. While on her round with her milk on Saturday morning, she called on Mrs. Arnold, and as she complained to her that she was suffering from diarrhœa, Mrs. Arnold gave her a dose of chlorodyne, which had as she thought been prepared for her son, but which it turned out was undiluted. On discovering her mistake, Mrs. Arnold sent for Saunders, and gave her some antimony as an antidote. She was left, however, to go on her way, and not returning home at her usual time, inquiry was made for her, and she was found, between 10 and 11 o'clock, in a water-closet in the town, in a state of unconsciousness. Medical attendance was called in, and every attention was shown her, but she never rallied, and only lingered till 10 o'clock at night. An inquest was held on Monday morning, when a verdict of "Accidental death from an overdose of chlorodyne" was rendered.—*London News.*

---

*The Cause of Scurvy.*—The general view that scurvy is produced by an excess of common salt in the blood, occasioned by a diet of salted meat exclusively, has received some confirmation, says the London Review, in the experiments lately conducted by M. Prussak of St. Petersburg. M. Prussak placed the webb of a frog's foot under the microscope, so as to observe the passage of the blood through the smallest blood-vessels. He then injected a solution of salt beneath the frog's skin, and watched the effect on the vessels. He perceived that the blood-corpuscles distended the vessels, and gave rise to the patches of dark-colored extravasations, extremely like the peculiar livid blotches seen on the skin of scorbutic patients. Experiments on dogs and other animals appear to give the same results. It now remains to be shown why common salt should possess this peculiar action on the blood vessels. Most probably the explanation will be found in the excessive osmosis which occurs owing to the increased density of the blood.

